# A Bomb under the Octopus

**Published:** 1906-04

**Authors:** 


### TEXAS MEDICAL JOURNAL.

AUSTIN, TEXAS.

A MONTHLY JOURNAL OF MEDICINE AND SURGERY.

EDITED AND PUBLISHED BY
F. E. DANIEL, M. D.
Mrs. F. E. DANIEL, Business Manager.

Published Monthly at Austin, Texas. Subscription price $1.00 a year in advance

### A BOMB UNDER THE OCTOPUS.

We have received a circular containing a letter and official order to all postmasters sent out by the Third Assistant Postmaster General, pointing out the abuse of the second-class mailing privileges by publications that charge either no subscription or a nominal one, and the circular shows, by the following, extracted from the *Journal* of the American Medical Association, that that publication is not a legitimate publication, entitled to second-class rates; and it will have to do one of two things: charge a subscription price and stick to it, or go out of business, as *the free distribution of any publication is a violation of the postal laws and must not only be stopped, but will be punished.* The circular states that *the same applies to all State journals* that are given free in consideration of payment of society dues. "Here's a pretty dow-de-do."

### MEMBERSHIP IN THE AMERICAN MEDICAL ASSOCIATION.

> The qualifications for membership require that the applicant be an active member of his county association, if there is one. If there is none, membership in the State Association, or in one of its branches, is necessary. Applications must be accompanied with a certificate showing that the applicant is an active member of such recognized society, and should be sent with the annual dues—five dollars—to the American Medical Association, 103 Dearborn Avenue, Chicago. ☞Members receive the *Journal* free. A subscriber who is qualified can have his name transferred to the membership list without expense, provided his subscription for the fiscal year is paid.—*Journal American Medical Association.*

"I have so lived that I have no fear of striking the briar-patch when I cross the divide." —*James Stephen Hogg.*

